# Examining equity in health insurance coverage: an analysis of Ghana’s National Health Insurance Scheme

**DOI:** 10.1186/s12939-018-0793-1

**Published:** 2018-06-18

**Authors:** Fidelia A. A. Dake

**Affiliations:** Regional Institute for Population Studies, University of Ghana, P.O. Box LG 96, Legon, Accra Ghana

**Keywords:** Universal health coverage, National Health Insurance Scheme, Ghana, Equity

## Abstract

**Background:**

Following years of out-of-pocket payment for healthcare, some countries in Africa including Ghana, Kenya and Rwanda have instituted social health protection programs through health insurance to provide access to quality and affordable healthcare especially for the poor. This paper examines equity in coverage under Ghana’s National Health Insurance Scheme (NHIS).

**Methods:**

Secondary data from the 2008 Ghana Demographic and Health Survey based on an analytical sample of 4821 females (15–49 years) and 4568 males (15–59 years) were analysed using descriptive, bivariate and multivariate methods. Concentration curves and indices were used to examine equity in coverage on the NHIS.

**Results:**

As at 2008, more than 60% of Ghanaians aged 15–59 years were not covered under the NHIS with slightly more females (38.9%) than males (29.7%) covered. Coverage was highest among the highly educated, professionals, those from households in the richest wealth quintile and urban residents. Lack of coverage was most concentrated among the poor.

**Conclusions:**

Universal coverage under the NHIS is far from being achieved with marked exclusion of the poor. There is the need for deliberate action to enrol the poor under the NHIS.

## Background

Achieving universal health coverage (UHC) is central to the next era of development frameworks including the Sustainable Development Goals (SDGs). SDG goal three (SDG 3) seeks to ensure healthy lives and promote well-being for all at all ages. A pivotal target of SDG 3 is to achieve UHC, including; financial risk protection, access to quality essential healthcare services and access to safe, effective, quality and affordable essential medicines and vaccines for all [1].

The World Health Organization defines UHC as “ensuring that all people have access to [promotive, preventive, curative, rehabilitative and palliative health services they need], of sufficient quality to be effective, while also ensuring that the use of these services does not expose the user to financial hardship” [2]. This definition embodies three key objectives: (i) equity in access to health services - those who need the services should get them, not only those who can pay for them; (ii) the quality of health services is good enough to improve the health of those receiving services; and (iii) financial-risk protection - ensuring that the cost of using care does not put people at risk of financial hardship. It has been emphasised that as countries seek to achieve UHC the first and primary objective of equity needs to be upheld [3, 4].

In response to the call to provide affordable health care for all, most low-and-middle income countries including some countries in Africa, e.g. Ghana, Kenya and Rwanda have instituted one form of social health protection program [5] or the other including via health insurance [3, 6] with the aim of achieving UHC [7]. Social health protection has been identified as a strategy for achieving universal access to healthcare by the International Labor Organisation who define social health protection as “a series of public or publicly organized and mandated private measures against social distress and economic loss caused by the reduction of productivity, stoppage or reduction of earnings or the cost of necessary treatment that can result from ill health” (ILO, 2008:3) [8]. Additionally, social health insurance is recognised as “a key element of social health protection and an integral means of achieving universal and affordable coverage” (Scheil-Adlung et al., 2006:14) [6]. Further to this, Scheil-Adlung et al. emphasize that “universal coverage needs to ensure access to care for every resident in a country [in order to be considered effective]” (Scheil-Adlung et al., 2006:14) [6].

According to O’Connell (2012) [6] Ghana, China, Rwanda and Vietnam have approached near universal access to a formally defined set of essential healthcare interventions. This notwithstanding, Ghana and India were also identified as making small but progressive steps towards full population coverage. Ghana’s National Health Insurance Scheme has been viewed as a model for other African countries because of the progress it has achieved in reaching a large section of the population in a short period of time since its implementation [10, 11].

However, these successes notwithstanding, Ghana still has a long way to go in achieving universal health coverage in its real sense. In this regard, there is a need for an equity analysis to identify population sub-groups who are at risk of being excluded as a necessary step towards achieving equity and universality. While there are several dimensions to equity, one critical but often overlooked dimension which is gender equity is necessary for achieving equity in UHC. Witter et al. (2017) [12] caution that the “movement towards UHC can fail to achieve gender balance or improve equity and may even exacerbate gender inequity” if not properly addressed. Thus in conducting research on equity in UCH, it is also important to investigate the component of gender equity but this has received limited attention in previous studies [13], especially for men, as the focus has mostly been on women and children.

Against the foregoing, this paper examines equity in coverage under the NHIS among the population aged 15–59 years (females; 15–49 years and males; 15–59 years). The next sections of the paper provide an overview of Ghana’s NHIS, the methodology employed in conducting the study, the results, discussion, conclusion and recommendation/policy implications.

### Ghana’s national health insurance scheme

Ghana is the first country in Africa to implement a National Health Insurance Scheme (NHIS) [14] to meet the needs of its population with the aim of achieving UHC [7]. Kotoh et al. (2018) [15] note that, the NHIS is “the first nationwide scheme in Africa initiated by a government”. The NHIS has long been awaited and has only recently come into being after many years of a system of out-of-pocket payment for healthcare known as the “cash and carry” system [16]. The NHIS was established by an act of parliament (Act 650 of 2003) to secure access to basic healthcare services to persons resident in the country through mutual and private health insurance schemes [17]. The NHIS became operational in 2005 following the passing of the necessary legislation in 2003. A governing body known as the National Health Insurance Authority (NHIA) was later established through Act 852 of 2012 to implement the NHIS [9]. The objective of the NHIA is to attain universal health insurance coverage in relation to: (a) persons resident in the country, and (b) persons not resident in the country but who are on a visit to the country, and to provide access to healthcare services to persons covered by the scheme. Among several other functions, the NHIA seeks to ensure: (i) equity in healthcare coverage (ii) access by the poor to healthcare services and (iii) protection of the poor and vulnerable against financial hardship [18].

The NHIS applies an out-of-pocket premium exemption policy as a means of ensuring that the poor and vulnerable have access to healthcare [15, 16, 19, 20]. Exempt groups include children under 18 years whose parents are covered under the scheme, those aged 70 years and above, pensioners under the social security pension scheme, persons classified as indigents (impoverished), and pregnant women (starting in July 2008) [16, 18, 21]. Formal sector workers who make contributions to the social security scheme pay 2.5% of their contributions as premium [15, 23]. The NHIS is financed through a hybrid mechanism which combines resource mobilization from taxes (2.5% value added tax on selected goods and services), pay roll deductions (2.5% of the 17.5% of formal sector workers’ Social Security and National Insurance Trust (SSNIT) contributions), government funding (an annual allocation of central government funds) and out-of-pocket payment of premiums by adults in the informal sector who are non-SSNIT contributors [15, 20]. The NHIA mandates a predefined benefits package that covers almost 95% of the disease burden in Ghana and includes in-patient hospital care, out-patient care at primary and secondary levels, and emergency and transfer services [16, 22]. NHIS subscribers can access healthcare from “all public, quasi-government, faith-based and some private health facilities [as well as] chemist shops and pharmacies that have been accredited and operate under contract with the NHIA” [15].

There has been significant gains in coverage under the NHIS since its establishment. The number of the people covered has increased from an initial 1.3 million in 2005 to about 8.8 million in 2012 [23]. Thus only about a third of the Ghanaian population are covered, leaving the remaining two-thirds exposed and vulnerable to catastrophic and impoverishing out-of-pocket payments. This level of coverage falls short of the national target of covering the entire population within five years of the implementation the NHIS [24] and the international goal of achieving UHC.

## Methods

### Source of data

This paper analyses nationally representative secondary data from the 2008 Ghana Demographic and Health Survey (GDHS). The Demographic and Health Survey (DHS) is a nationally representative survey that is conducted every five years to assess demographic and health outcomes in developing countries. The DHS includes core modules on fertility, contraceptive use and nuptiality among others as well as country specific modules on different interventions. The 2008 round of the GDHS included a national module in which myriad national policies including the NHIS were assessed. This paper combines data from questions on coverage under NHIS with demographic and socio-economic indicators and lifestyle risk factors to examine equity in NHIS coverage. The paper uses data from the 2008 round of the GDHS because this is the closest to the five year target of attaining UHC in Ghana following implementation of the program in 2005. The paper uses data from the female and male data files, the analyses and results are thus stratified by sex.

### Study participants

The participants in this study include females aged 15–49 years and males aged 15–59 years who were interviewed in the 2008 round of the GDHS. The participants were selected through a two-stage sampling design. The first stage of sampling involved the selection of a total of 412 clusters nationwide. At the second stage of sampling, 30 households were selected from each of the previously selected clusters. Females aged 15–49 years and males aged 15–59 years in the selected households were eligible to be interviewed for the survey if they were usual residents or visitors who were present in the household on the night preceding the survey. A total of 5096 females and 4769 males were identified as being eligible to be interviewed and out of these, 4916 females and 4568 males were interviewed. The analytical sample for this study consists of 4821 and 4568 (weighted sample) females and males respectively who had valid data on the variables used in the analysis.

### Variables

#### Dependent variable

The dependent variable for this study was measured using participants’ coverage under the NHIS. The dependent variable was measured using the question “What type of health insurance do you have?” Those who indicated that they had national/district health insurance were considered as being covered and vice versa. Thus participants’ NHIS status was categorised as a dichotomous variable; “*Covered”* if the participant reported being covered under the NHIS and “*Not covered”* if the participant reported not being covered under the NHIS.

#### Independent variables

The characteristics of the participants that could influence their being enrolled on the health insurance scheme were assessed. These include their demographic characteristics such as age, marital status and parity (for females); socio-economic characteristics including highest level of education attained and type of occupation; geographic factors including place and region of residence and lifestyle risk factors including physical activity, alcohol consumption and body mass index (for females). Key household characteristics including household size and wealth status were also controlled for. The age of respondents’ was categorised into five year age groups while marital status had three categories of never married, married or in union and formerly married including those who were widowed, divorced or separated. Parity was applicable to only females and was assessed as the number of children a woman has given birth to and used as a continuous variable. The highest level of education attained by respondents had five categories of no formal education, primary, middle, secondary and tertiary level. There were five occupational groups: no occupation, professionals, sales/service personnel, agricultural and manual workers. Additionally, unhealthy behaviours such as alcohol consumption and physical inactivity which constitute risk factors for myriad lifestyle diseases with relatively high healthcare costs were included in the analysis. Alcohol consumption was assessed based on whether respondents consumed alcohol or not. Physical activity was measured as the number of days in the last seven days (immediately preceding the survey) respondents’ engaged in vigorous physical activity that lasted for at least 15 min. The measure of physical activity was used as a continuous variable. Body mass index which was obtained by dividing the weight (in kilograms) of respondents’ by their height (in meters squared) was available for females but not males and was used as a continuous variable.

### Methods of analysis

The characteristics of the study sample were described using frequencies and percentages. The distribution of the study sample by NHIS status and some key socio-demographic characteristics was assessed using chi-square analysis. The likelihood of the participants being covered under the NHIS was assessed using binary logistic regression analysis. Concentration curves and indices were used to examine equity in NHIS coverage. Household wealth status served as the living standards variable while NHIS status was used as the health variable in the equity analysis. The concentration curves were generated using Microsoft Excel 2013 while the statistical analyses were performed using IBM SPSS Statistics version 22.0.

## Results

The results show that as at 2008, more than 60 % of the Ghanaian population aged 15–59 years were not covered under the NHIS (Table 1). The results further revealed that a higher proportion of females (38.9%) compared to males (29.7%) were covered while the reverse is true in terms of no coverage (61.1 and 70.3% respectively). Regarding the demographic characteristics of the study sample, respondents’ aged 15–19 years constituted about one-fifth (females – 20.5%, males – 19.9%) of the total sample and more than half of both females and males were married or in union (58.8 and 54.6% respectively). Females had a slightly larger household size on average compared to their male counterparts and the females reported having had about 2 children on average. In terms of the level of education attained, a higher proportion of females (21.2%) compared to males (14.3%) had no formal education while 9% of males and about 4% of females had completed tertiary/higher level of education. Similarly, in terms of occupation, about thrice as many males (17.0%) as females (5.0%) were involved in professional/technical/managerial types of occupation while about 2 in 5 females (39.9%) compared to 6.6% of their male counterparts were engaged in the sales/services sector. There were relatively higher proportions of respondents belonging to the richer and richest wealth quintiles compared to the middle, poorer and poorest quintiles (Table 1). More than half of the study sample (females – 51.5%, males – 53.5%) resided in rural areas while the regional distribution shows respondents from the Ashanti region constituting the highest proportion followed by those from the Greater Accra region. The lifestyle behaviours of the respondents showed males reporting more number of physical activity days than their female counterparts but twice as many males (36.7%) compared to females (17.5%) reporting that they consume alcohol. The average body mass index of the female respondents was 23.6 kg/m^2^ (Table 1).

**Table 1:** Distribution of study sample by NHIS status, selected socio-demographic characteristics and lifestyle behaviours

Variable	Percentage (n) / Mean ± Standard deviation	
	Females	Males
NHIS Status		
Covered	38.9 (1877)	29.7 (1355)
Not covered	61.1 (2944)	70.3 (3213)
Socio-demographic characteristics		
Age		
15–19	20.5 (990)	19.9 (911)
20–24	18.0 (868)	15.4 (704)
25–29	17.0 (819)	13.7 (624)
30–34	13.2 (636)	11.7 (533)
35–39	13.1 (630)	11.6 (528)
40–44	9.5 (458)	8.6 (394)
45–49	8.7 (419)	8.0 (364)
50–54	na	6.5 (297)
55–59	na	4.7 (213)
Parity	2.33 ± 2.46	na
Household size	5.08 ± 2.69	4.55 ± 2.86
Marital status		
Never married	32.2 (1550)	42.5 (1942)
Married/In union	58.8 (2832)	54.6 (2404)
Formerly married	9.1 (438)	4.8 (221)
Highest level of education attained		
No formal education	21.2 (1023)	14.3 (652)
Primary	20.2 (972)	14.5 (661)
Middle	41.5 (2001)	42.6 (1947)
Secondary	13.2 (638)	19.7 (898)
Tertiary/Higher	3.9 (187)	9.0 (410)
Type of occupation		
Not working	23.2 (1117)	21.6 (988)
Professional/Technical/Managerial	5.0 (240)	17.0 (775)
Sales/Services	39.9 (1925)	6.6 (302)
Agricultural	23.4 (1127)	34.3 (1567)
Manual	8.5 (411)	20.5 (935)
Wealth status		
Poorest	15.8 (759)	17.7 (809)
Poorer	18.2 (877)	17.8 (815)
Middle	19.9 (961)	17.2 (784)
Richer	23.0 (1109)	23.6 (1079)
Richest	23.1 (1114)	23.7 (1081)
Place of residence		
Urban	48.5 (2340)	46.5 (2125)
Rural	51.5 (2481)	53.5 (2443)
Region of residence		
Western	9.2 (444)	10.5 (479)
Central	8.6 (416)	8.2 (376)
Greater Accra	17.3 (832)	16.1 (735)
Volta	8.7 (419)	9.2 (419)
Eastern	9.8 (474)	10.3 (470)
Ashanti	20.7 (1000)	18.8 (857)
Brong Ahafo	8.8 (423)	8.4 (385)
Northern	9.5 (456)	10.4 (477)
Upper East	4.9 (237)	5.5 (249)
Upper West	2.5 (120)	2.6 (120)
Lifestyle risk factors		
Alcohol consumption		
Yes	17.5 (844)	36.7 (2890)
No	82.5 (3977)	63.3 (2890)
Physical activity	1.74 ± 2.38	2.93 ± 2.60
Body mass index (kg/m^2^)	23.6 ± 4.80	na
Total (N)	100.0 (4821)	100.0 (4658)

*na* not applicable

The bivariate analysis does not show a clearly discernible age pattern except that NHIS coverage was highest among females in the 40–44 age group and males in the 55–59 age group (43.1 and 39.0% respectively) (Table 2). Again, coverage was observed to be highest among those who were married (females – 41.4%, males – 31.7%) but less common among those who were formerly married, particularly males (82.8%). The results further show that among both females and males, coverage under the NHIS was highest among those with tertiary/higher education (54.5 and 56.3% respectively), professionals including technical and managerial workers (56.2 and 44.9% respectively), those from households in the richest wealth quintile (47.0 and 39.9% respectively) and urban residents (41.8 and 34.6% respectively) (Table 2). Among the ten administrative regions, coverage was highest in the Brong Ahafo region for both females (58.9%) and males (44.4%) and lowest in the Central and Greater Accra regions for females (23.0%) and males (21.7%) respectively.

**Table 2:** Percentage distribution of study sample by selected socio-demographic characteristics and NHIS status among females and males

Socio-demographic characteristics	NHIS Status			
	Covered		Not covered	
Age	Females	Males	Females	Males
15–19	37.1	33.8	62.9	66.2
20–24	33.8	22.6	66.2	77.4
25–29	40.4	20.5	59.6	79.5
30–34	42.0	34.0	58.0	66.0
35–39	42.2	30.8	57.8	69.2
40–44	43.1	30.7	56.9	69.3
45–49	37.0	28.0	63.0	72.0
50–54	na	37.2	na	62.8
55–59	na	39.0	na	61.0
Marital status				
Never married	36.0	28.6	64.0	71.4
Married/In union	41.4	31.7	58.6	68.3
Formerly married	33.1	17.2	66.9	82.8
Highest level of education attained				
No formal education	32.7	18.6	67.3	81.4
Primary	30.0	21.8	70.0	78.2
Middle	42.3	27.1	57.7	72.9
Secondary	47.3	36.9	52.7	63.1
Tertiary/Higher	54.5	56.3	45.5	43.7
Type of occupation				
Not working	39.3	34.3	60.7	65.7
Professional/Technical/Managerial	56.2	44.9	43.8	55.1
Sales/Services	39.4	25.5	60.6	74.5
Agricultural	31.0	22.4	69.0	77.6
Manual	47.4	25.5	52.6	74.5
Wealth status				
Poorest	29.5	17.6	70.5	82.4
Poorer	32.2	22.7	67.8	77.3
Middle	37.4	26.8	62.6	73.2
Richer	44.0	35.8	56.0	64.2
Richest	46.9	39.9	53.1	60.1
Place of residence				
Urban	41.8	34.6	58.2	65.4
Rural	36.2	25.3	63.8	74.7
Region of residence				
Western	42.6	30.7	57.4	69.3
Central	23.1	23.9	76.9	76.1
Greater Accra	24.5	21.7	75.5	78.3
Volta	30.1	24.3	69.9	75.7
Eastern	50.1	36.6	49.9	63.4
Ashanti	40.8	28.1	59.2	71.9
Brong Ahafo	58.9	44.4	41.1	55.6
Northern	39.5	31.2	60.5	68.8
Upper East	55.3	29.2	44.7	70.8
Upper West	47.1	43.3	52.9	56.7
Total	38.9	29.7	61.1	70.3

*na* not applicable

The results of the multivariate logistic regression analysis show that among males, those aged 20–49 years were significantly less likely to be covered under the NHIS compared to those aged 55–59 (Table 3). Among both females and males, increasing household size was associated with a higher likelihood of being covered under the NHIS. Furthermore, the results revealed disparities in socio-economic status and NHIS coverage. For example, compared to those with tertiary/higher education, those at the lower ends of the education spectrum were significantly less likely to be covered under the NHIS (Table 3). Among males, those who have completed various levels of education other than tertiary were significantly less likely to be covered while among females, those with no formal education and those with primary education were significantly less likely to be covered compared to those with higher education. With regards to occupational status, male professionals were significantly more likely to be covered compared to their counterparts who were manual workers while among females, those in the sales/services sector and those in agriculture were significantly less likely to be covered compared to their counterparts who were manual workers. The results also showed marked disparities in NHIS coverage by wealth status. The likelihood of being covered under the NHIS was significantly lower for all wealth status categories other than the richest with those from the poorest households being the most disadvantaged (Table 3). The different lifestyle risk factors controlled for were also found to be significantly associated with the likelihood of being covered under the NHIS. Among males, increased involvement in vigorous physical activity and non-consumption of alcohol were associated with increased odds of being covered while among females, each additional increase in BMI was associated with an increased likelihood of being covered under the NHIS (Table 3).

**Table 3:** Results of a binary logistic analysis predicting the likelihood of coverage under NHIS

	Odd ratios	
	Women	Men
Demographic characteristics		
Age ^[women-45-49,men-55-59]^	1.000	1.000
15–19	1.030	0.746
20–24	0.727^+^	0.339***
25–29	0.937	0.357***
30–34	1.070	0.703^+^
35–39	1.210	0.687*
40–44	1.296^+^	0.714^+^
45–49	na	0.659*
50–54	na	0.902
Parity	0.978	na
Household size	1.025^+^	1.048**
Marital status ^[Formerly married]^	1.000	1.000
Never married	0.874	1.438
Married/In union	1.502**	1.778**
Socio-economic characteristics		
Highest level of education attained ^[Tertiary]^	1.000	1.000
No formal education	0.507**	0.251***
Primary	0.494**	0.310***
Middle	0.827	0.391***
Secondary	1.119	0.617**
Type of occupation ^[Manual worker]^	1.000	1.000
Not working	0.787^+^	1.579***
Professional/Technical/Managerial	1.023	1.802***
Sales/Services	0.623***	0.910
Agricultural	0.556***	1.167
Wealth status ^[Richest]^	1.000	1.000
Poorest	0.166***	0.136***
Poorer	0.299***	0.252***
Middle	0.416***	0.358***
Richer	0.627***	0.647***
Geographic factors		
Place of residence ^[Rural]^	1.000	1.000
Urban	0.799*	0.855
Region of residence ^[Greater Accra]^	1.000	1.000
Western	4.327***	2.655***
Central	1.746***	2.126***
Volta	3.094***	2.783***
Eastern	6.789***	4.908***
Ashanti	3.808***	2.606***
Brong Ahafo	13.187***	9.472***
Northern	7.900**	7.271***
Upper East	18.069***	7.854***
Upper West	10.800***	12.473***
Lifestyle risk factors		
Physical activity	1.008	1.006
Body mass index	1.025**	na
Alcohol consumption ^[Yes]^		
No	1.072	1.251**

^+^*P* < 0.10 *P < 0.05 **P < 0.01 ***P < 0.001

*na* not applicable

**Fig. 1:**
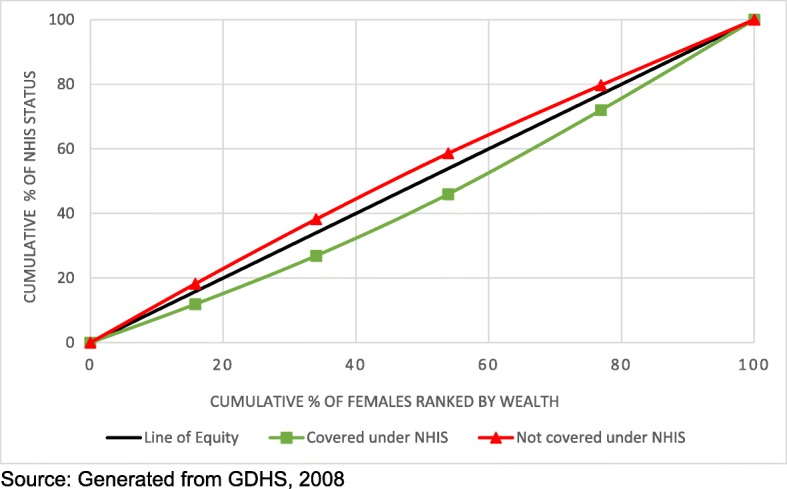
Concentration curve showing wealth disparities in NHIS coverage among females

**Fig. 2:**
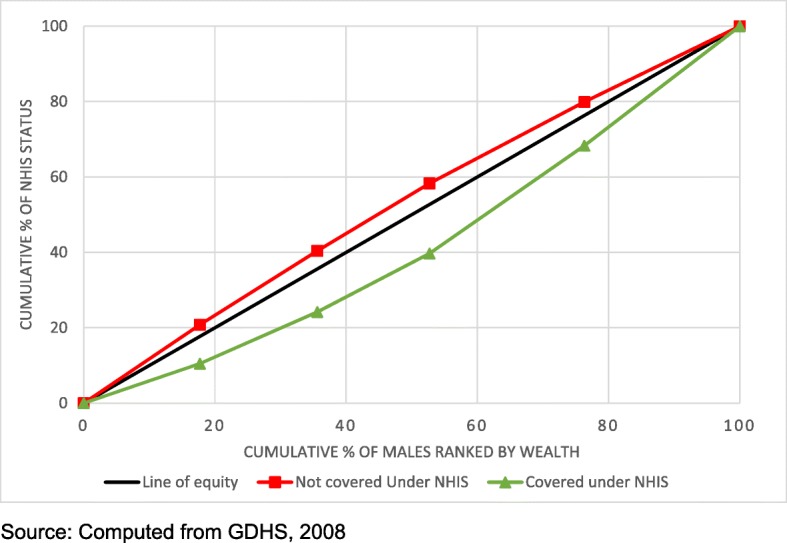
Concentration curve showing wealth disparities in NHIS coverage among males

The concentration curves reveal inequity in NHIS coverage. Figures 1 and 2 show that lack of coverage under the NHIS was more concentrated among the poor while coverage was more concentrated among the rich for both females and males. The concentration indices shown in Table 4 also show that lack of coverage under the NHIS was most concentrated among the poor with men being more affected compared to women.

**Table 4:** Concentration indices showing inequity in health insurance coverage by wealth groups among women and men

	Concentration Index	
Wealth groups	Women	Men
Poorest	−0.00152	−0.00254
Poorer	−0.00666	−0.00536
Middle	−0.02105	−0.02376
Richer	−0.00280	−0.03600
Richest	0.00000	0.00000
Total	−0.03203	−0.06766

## Discussion

The results of this study demonstrate that coverage under Ghana’s NHIS is low with just about one-third of the population aged 15–59 years being covered in 2008. Coverage has continued to remain low and the NHIA reports that only 34% of the population was enrolled in 2011, a year after the 2010 target of attaining universal coverage. Findings from other studies corroborate this. For example, NHIS enrolment in an urban and rural district in one region in Southern Ghana between 2010 and 2013 was found to be between 30 and 40% and the authors further note that new enrolments over the period appear to have stalled [20]. In other related studies, Kotoh and Van der Geest (2016) [25] found that just about 40% of 6790 individuals in fifteen communities in the Central and Eastern regions were insured while Sarpong et al. (2010) [26] found that only 38% of 7223 households in the Asante Akim North district in the Ashanti region were NHIS subscribers and Kusi et al. (2015) [27] using cross-sectional data from three districts representative of the three ecological zones of Ghana found that just about 28% of surveyed households were fully insured members of the NHIS. These findings are closely related to rural urban and regional disparities in NHIS coverage as found in the current study and other studies. In the current study, respondents from all regions except Greater Accra were more likely to be covered under the NHIS with the odds being highest for those in the three northern regions and the Brong Ahafo, Ashanti and Eastern regions. This finding is similar to those of Amu and Dickson (2016) [22] who found that women in the savannah and forest zones of Ghana were more likely to have health insurance compared to those in the coastal zone.^1^ Similarly, Dixon et al. (2011) [28], using the 2008 GDHS data found inequity in coverage between the Northern and Southern half of the country. Dixon et al. (2011) [28] also note that one of the plausible reasons for the observed regional disparities in NHIS coverage is possibly because the northern regions have a prior history of community-based insurance schemes. Additionally, the northern half of Ghana is known to be relatively poorer compared to the Southern half and it has been noted that in such contexts, having health insurance is an affordable way to access healthcare rather than paying out of pocket.

The findings also revealed that a higher proportion of women (38.9%) compared to men (29.7%) were covered under the NHIS which corroborates the findings of Dixon et al. (2011) [28]. Additionally, Kotoh et al. (2018) [15] in their study on factors that influence enrolment and retention in the NHIS in the Central and Eastern regions of Ghana, found that females constituted more than half of those who were covered. One of the likely reasons for this gender difference is that women tend to report poorer health status compared to men [29, 30]. Women are thus more likely to need healthcare, explaining why they were more likely to be covered. Women also tend to use healthcare services more often than men, probably explaining why a higher proportion of women compared to men were covered under the NHIS. Additionally, the cost of healthcare has been found to be higher among women than men [31]; the higher cost of seeking health care for women is a likely explanation for why a higher proportion of women than men were covered under the NHIS. Also, it is argued that males, particularly wealthy Ghanaian males may have the means to afford alternative options of health financing including from private providers [10] which often tends to be more expensive, whereas females may not be able to afford these alternative options. Furthermore, given the caregiving roles of women in the household, having health insurance including for themselves and their children gives women a means accessing healthcare without the burden of having to pay out-of-pocket [10, 32].

The analyses also reveal inequities in coverage under Ghana’s NHIS. Inequity was observed for key socio-economic status variables including level of education, type of occupation and wealth status. Among men, all levels of education other than tertiary were associated with a lower likelihood of coverage under the NHIS. Among women, no formal education and primary level of education were also associated with a lower likelihood of coverage under NHIS. Similar findings regarding level of educational attainment and NHIS status have been reported in several other studies [10, 21, 22, 33]. Similarly, inequity in coverage across wealth quintiles was clearly evident, with the poorest (both women and men) being most disadvantaged, highlighting the exclusion of the poor from NHIS coverage. Findings from other studies [4, 10, 15, 33] corroborate this socio-economic inequity in NHIS coverage. These findings reinforce the relationship between education, occupation and wealth status. People who are highly educated tend to understand the importance of having insurance and they also tend to have the ability to purchase health insurance and the rich tend to purchase health insurance even if they do not need it [22].

The findings of this study indicate that although Ghana’s NHIS was intended to be a pro-poor initiative [4], this is not the case in reality. The richest are more likely to be covered and are thus more likely to benefit from the associated financial risk protection while the poor are less likely to be covered, exposing them to possible financial hardships associated with having to make out-of-pocket payments for healthcare. These findings imply that even though Ghana may be making progressive steps towards full population coverage, this aim is far from being achieved given that more than 60% of the population aged 15–59 years were not covered under the NHIS as at 2008. Furthermore, indications from projections suggest that attaining UHC in Ghana in its true sense will take nearly six decades to happen in 2076 [34]. It is therefore important that the government of Ghana takes deliberate steps to ensure that the poor in particular are enrolled on the NHIS. There may be the need for reforms similar to what was done in Rwanda with the introduction of Mutuelles de Sante which achieved more than 90% coverage over a decade [35, 36]. Additionally, different mechanisms may be required in identifying and enrolling the poor on the NHIS. In other sub-Saharan African countries such as Burkina Faso, community wealth ranking has been used to identify the poorest households and such households receive subsidies to enable them enrol in community-based health insurances schemes [35].

### Strengths and limitations

This study is one of the few studies that conducts an equity analysis on NHIS coverage among both women and men using nationally representative data. The study is, however, not without a number of limitations. Firstly, the study uses data from the 2008 GDHS and not the recent 2014 GDHS because the 2008 data is closest to the year of implementation of the program and provides a much closer timeline to the set target of reaching universal population coverage five years after implementation of the program. One limitation though is that 2008 is two years earlier than the five year target which would have been in 2010 but again the 2014 data is also quite far away from the 2010 target. Thus even though 2008 GDHS is an older data set, it is more appropriate for this particular study. Secondly, as this study uses data from a secondary source, there were limitations in terms of some variables not being available e.g. BMI was only available for females. Additionally, the age range differed for males and females because of the difference in the age eligibility criteria used for selecting respondents for the demographic and health survey. The analyses were thus conducted using different age limits for females and males as was available in the data and without the inclusion of BMI for males. These limitations notwithstanding, the results from the current analyses are valid.

## Conclusion

This study provides empirical evidence of initial progress towards UHC in Ghana following the implementation of the NHIS in 2005. The findings indicate that, before Ghana can achieve UHC, inequities against the poor would have to be critically and effectively addressed. Additionally, unless coverage rates are increased and the poor are reached, universality and equity in coverage under the NHIS cannot be achieved. The government of Ghana therefore needs to take deliberate action to properly identify and enrol poor, vulnerable and marginalized groups on the NHIS. It is also imperative that the country continuously monitors progress towards achieving UHC if the goal of universal population coverage on the NHIS is to be achieved.

## Abbreviations

DHS: Demographic and Health Survey
GDHS: Ghana Demographic and Health Survey
NHIA: National Health Insurance Authority
NHIS: National Health Insurance Scheme
SDGs: Sustainable Development Goals
SSNIT: Social Security and National Insurance Trust
UHC: Universal Health Coverage

## Footnotes

1 Amu and Dickson (2016) recoded the ten administrative regions into general ecological zones. The classification of administrative regions into ecological zones were done as follows; Northern, Upper West and Upper East regions – Savannah zone, Brong Ahafo, Ashanti and Eastern regions – Forest zone and Western, Central, Greater Accra and Volta regions – Coastal zone.
